# Molecular interactions, solubility, and cytotoxic activity of 4‑hydroxycoumarin in choline chloride‑based acidic deep eutectic solvents

**DOI:** 10.1038/s41598-026-56725-w

**Published:** 2026-06-15

**Authors:** Mohammad Khorsandi, Hemayat Shekaari, Masoumeh Mokhtarpour, Parisa Akbarzadeh Gondoghdi, Hamed Hamishehkar

**Affiliations:** 1 Department of Physical Chemistry, University of Tabriz, Tabriz, Iran; 2 Drug Applied Research Center, Tabriz University of Medical Sciences, Tabriz, Iran; 3 New Material and Green Chemistry Research Center, Khazar University, 41 Mehseti Street, Baku, AZ1096 Azerbaijan

**Keywords:** Thermodynamic modeling, Hansen solubility parameters, ChCl/Pro, Drug solubility, MTT assay, Green pharmaceutical solvents, Biochemistry, Cancer, Chemistry, Drug discovery

## Abstract

This study systematically investigated the solubility enhancement of 4-hydroxycoumarin (4HC), a poorly water-soluble pharmaceutical compound, in three novel acidic deep eutectic solvents (ADESs) prepared from choline chloride (ChCl) and acidic hydrogen bond donors encompassing propionic acid (Pro), acetic acid (Ace) and lactic acid (Lac). The solubility measurements were conducted Appling the shake-flask method at 298.15–313.15 K. The ChCl/Pro system exhibited unprecedented more than 500-fold solubility improvement over the pure water, with solubility Increasing in the order: ChCl/Pro > ChCl/Ace > ChCl/Lac. Hansen Solubility Parameters (HSPs) analysis confirmed hydrogen bonding and acidity as dominant dissolution factors. In addition, advanced thermodynamic models (Wilson, e-NRTL, UNIQUAC) accurately correlated experimental solubility data, with the Wilson model achieving ARD percentage values as low as 0.10%. Thermodynamic analysis revealed the dissolution process is endothermic and enthalpy-driven. MTT cytotoxicity assays on HT29 colon cancer cells demonstrated IC₅₀ values in the 20–100 µg/mL range, with differential cytotoxicity correlated to solvent composition. These findings identify ChCl/Pro as the optimal ADES candidate (500-fold solubility enhancement, IC_50_ 20–100 µg/mL range) for preformulation development of 4HC, establishing a data-driven foundation for green solvent-based pharmaceutical applications.

**Supplementary Information:**

The online version contains supplementary material available at https://doi.org/10.1038/s41598-026-56725-w.

## Introduction

Poorly soluble drugs represent a persistent challenge in pharmaceutical development, with inadequate aqueous solubility critically limiting bioavailability and therapeutic efficacy^1^. Contemporary estimates suggest that approximately 60–90% of newly synthesized drug candidates exhibit poor water solubility, necessitating innovative formulation strategies. Among naturally occurring bioactive compounds, coumarin derivatives demonstrate remarkable pharmacological versatility, encompassing antioxidant, anticoagulant, anti-inflammatory, and anticancer properties^2,3^. Specifically, 4 hydroxycoumarin (4HC), a fundamental structural scaffold underlying warfarin and other clinically relevant anticoagulants, exhibits pronounced biological activity through protein interaction mechanisms, with recent studies emphasizing its dual antioxidant and pro-oxidant capabilities in modulating oxidative stress for selective cancer cell induction^4–6^. However, The 4HC is extremely limited aqueous solubility fundamentally restricts pharmaceutical applications and necessitates enhancement through rational solvent design^7,8^.

The framework of sustainable pharmaceutical development increasingly calls for the replacement of conventional organic solvents, which are characteristically flammable, volatile, and environmentally harmful. On the flip side, deep eutectic solvents (DESs) have appeared as promising green chemistry alternatives, representing low-melting-point binary or ternary mixtures of hydrogen bond donors (HBDs) and acceptors (HBAs) that exhibit exceptional biodegradability, tunability, and low toxicity^9,10^. Recent pharmacokinetic studies have shown that appropriately designed DES formulations exhibit minimal cytotoxicity, positioning them as viable candidates for pharmaceutical applications^11,12^. The designation of DESs as therapeutic deep eutectic systems reflects their strong potential to enhance the solubility and permeability of active pharmaceutical ingredients (APIs) while improving bioavailability through rational composition optimization. Among DES variants, acidic deep eutectic solvents (ADESs) composed of organic acids as HBDs and quaternary ammonium salts, particularly choline chloride, as HBAs demonstrate enhanced solubilizing capacity through synergistic ion-dipole, dipole-dipole, and hydrogen-bonding interactions^13,14^. Moreover, recent advances have shown that natural DESs (NADESs) and acidic variants offer intrinsic advantages in biocompatibility and recyclability, making them particularly suitable for green polymeric and pharmaceutical material engineering applications^15^.

Quantitative prediction of drug solubility in alternative solvents requires rigorous integration of computational and experimental methodologies. Hansen solubility parameters (HSPs), a three-dimensional descriptor system encompassing dispersive, polar, and hydrogen-bonding interactions, enable rational a priori solvent screening without exhaustive experimental iteration^16,17^. Contemporary studies show that HSP analysis, when coupled with molecular simulation methodologies, provides superior discriminatory power for polymeric carrier selection and drug-solvent miscibility prediction compared with classical approaches^18^. Concurrently, advanced thermodynamic models based on local-composition frameworks (Wilson, e-NRTL, and UNIQUAC) provide mechanistic insight into activity-coefficient behavior across compositionally heterogeneous systems, facilitating predictive formulation design and improving the correlation of experimental solubility data^19–21^. In addition, thermodynamic analysis With van’t Hoff and Gibbs equations elucidates whether solubilization processes are enthalpy- or entropy-driven, with implications for thermal stability and storage conditions^22^.

Beyond solubility optimization, comprehensive risk characterization through in vitro cytotoxicity assessment remains essential for pharmaceutical viability. MTT (3-(4,5-dimethylthiazol-2-yl)-2,5-diphenyltetrazolium bromide)-based cell viability assays quantify IC_50_ values—the concentration required to induce 50% cell death—thereby establishing biocompatibility thresholds and revealing differential cellular responses to ADESs-drug formulations. Recent investigations utilizing MTT assay in multiple colon cancer models have reported composition-dependent cytotoxicity, in which the identity and ratio of HBD components modulate pharmacological potency through distinct effects on metabolic pathways, oxidative stress, and apoptotic cascade activation. Notably, coumarin derivatives have shown pronounced differential cytotoxicity against cancer cell lines such as MCF-7, HeLa, and HepG2, with IC50 values in the 2.54–8.57 µM range, outperforming camptothecin (CPT) while maintaining minimal toxicity toward normal kidney cells (LLCPK1, IC50 > 94 µM)^23–26^.

These composition-tunable properties indicate that choline chloride-based acidic deep eutectic solvents (ADESs) can prove as promising pharmaceutical excipients, capable of enhancing the apparent solubility of poorly water-soluble drugs such as 4HC while providing a biocompatible microenvironment for controlled drug release, thereby supporting the development of greener and more sustainable dosage forms. In this context, 4-hydroxycoumarin represents an attractive model scaffold for preformulation studies, as improved solubilization and acceptable biocompatibility are prerequisites for any future therapeutic application. Therefore, a systematic characterization of it’s solubility, molecular interactions, and in vitro cytotoxicity in choline chloride-based ADESs is directly relevant to rational formulation and drugdelivery design.

The present investigation systematically addresses this knowledge gap by simultaneously evaluating the solubility enhancement of 4HC across three structurally distinct ADESs (ChCl/Pro, ChCl/Ace, and ChCl/Lac). In the second step, quantitative solubility prediction was performed via HSP analysis and rigorous thermodynamic correlation using three local-composition models. This was followed by mechanistic thermodynamic characterization to elucidate enthalpy-entropy contributions through van’t Hoff analysis. Eventually, differential cytotoxic responses in HT29 cells were assessed via the MTT assay, thereby providing integrated biochemical and toxicological data supporting rational ADESs-based pharmaceutical formulation design aligned with green chemistry principles and advancing the paradigm of sustainable pharmaceutical manufacturing.

## Laboratory section

### Materials

Analytical-grade reagents employed in this investigation were procured from internationally recognized commercial suppliers, ensuring ≥ 99.5% mass fraction purity as verified by supplier certificates of analysis (CoA). Ethanol (CAS 64-17-5, ≥ 99.8%) was sourced from Sigma-Aldrich (St. Louis, MO, USA), while propionic acid (CAS 79-09-4), choline chloride (ChCl, CAS 67-48-1), lactic acid (CAS 50-21-5), acetic acid (CAS 64-19-7), and 4-hydroxycoumarin (4HC, CAS 107-02-8) were obtained from Merck KGaA (Darmstadt, Germany). All chemicals were employed as received without further purification, consistent with contemporary green chemistry protocols that minimize energy-intensive processing. Ultrapure water was prepared in-house using a Milli-Q^®^ Direct 8 system (Merck Millipore, Burlington, MA, USA), incorporating double distillation, deionization (resistivity 18.2 MΩ·cm at 25 °C), and 0.22 μm filtration. Detailed physicochemical specifications—including CAS registry numbers, molecular formulae, and molar masses—are systematically compiled in Table 1.

**Table 1:**
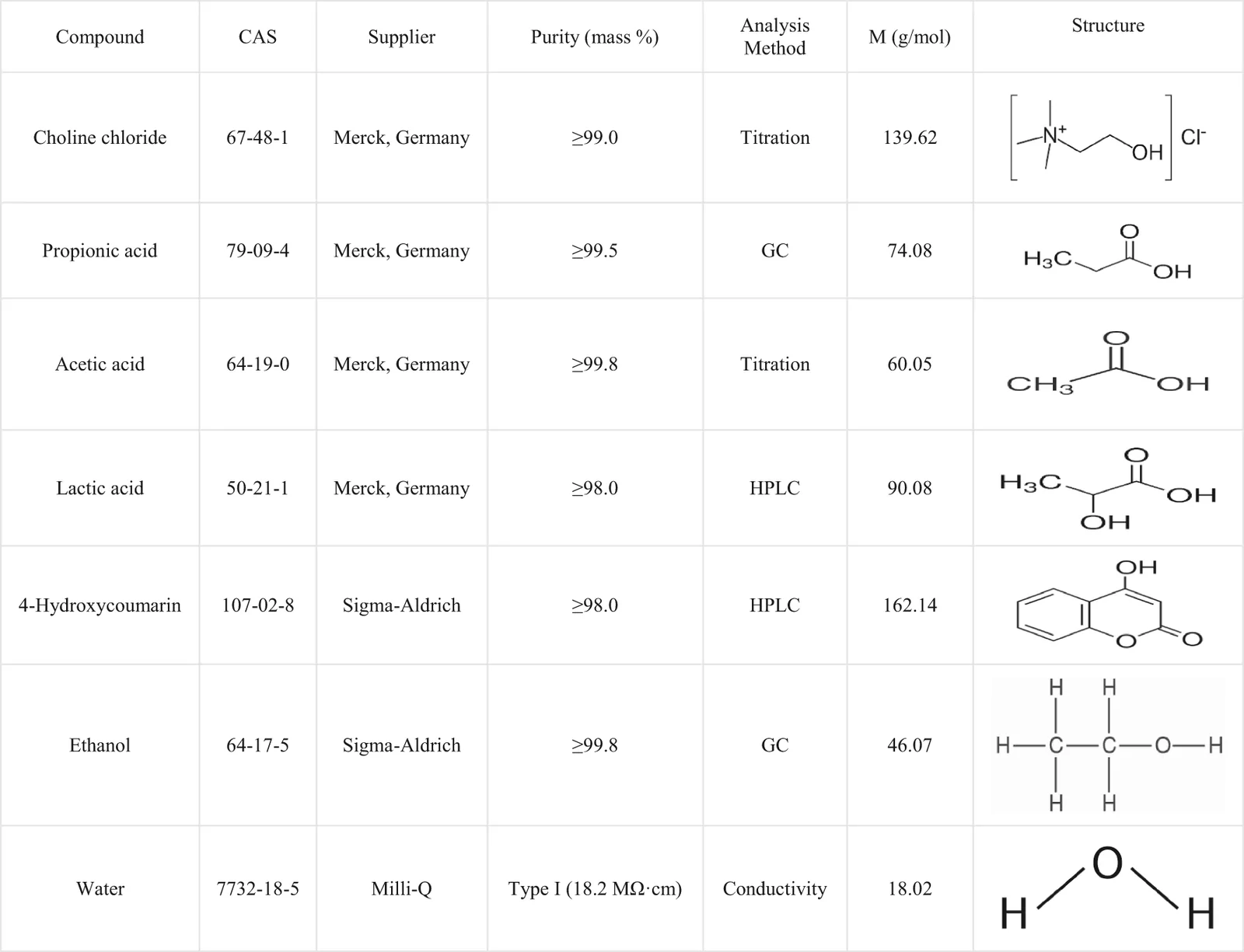
Descriptions of the compounds used, as well as the CAS number of the supplier, purity, and chemical structure of the substance.

### Preparation of Acidic Deep Eutectic Solvents (ADESs)

The ADESs in this investigation were prepared following established protocols with minor modifications for enhanced reproducibility. Choline chloride (ChCl) as the hydrogen bond acceptor (HBA) was combined with individual hydrogen bond donors (HBDs)—propionic acid (PA), acetic acid (AA), or L-(+)-lactic acid (LA)—at a fixed 1:2 molar ratio (HBD: ChCl)^27,28^. Components were weighed using an analytical balance (AW220, Shimadzu, Japan; readability ± 0.1 mg) and transferred to clean, dry glass vials^29^.

The mixtures were subjected to magnetic stirring (500 rpm) at 90 °C (363.15 K) for 2 h in a temperature-controlled oil bath until complete homogenization was achieved, as evidenced by formation of a transparent, colorless liquid free of undissolved solids^30^. Post-preparation, ADESs were dried under reduced pressure (0.1 mbar) at 25 °C for 24 h using a rotary vacuum evaporator to minimize residual water content and enhance long-term stability. Water content in the final ADESs formulations was quantified by Karl Fischer volumetric titration (751 GPD Titrino, Metrohm, Switzerland) employing a double platinum electrode and CombiTitrant 5 reagent. Triplicate measurements confirmed moisture levels below 0.02 wt% (200 ppm), satisfying pharmacopeial requirements for solvent-grade DESs. It is nottable that physicochemical properties including molar ratio, molar weight, acidity, and water content are summarized in Table 2.

**Table 2:** Common features of ADESs employed in this research at 298.15 K and 866 hPa.

ADESs	Salt - HBD (molar ratio)	M_ADES_ (g mol^-1^)	Acidity (α = pKa)	Water content (wt%)
ChCl / Ace	1.00:2.00^b^	95.93	4.87	0.02
ChCl / Pro	1.00:2.00	86.58	4.76	0.02
ChCl / Lac	1.00:2.00	106.59	3.86	0.03

Standard uncertainty for u*(T)* = 0.1 K and u*(P)* = 10 hPa.

^b^ Standard uncertainty (u) was calculated to be smaller than 0.05 mol ratios for ADESs composition.

### Hansen solubility parameters (HSP)

The choice of a suitable solvent is a critical step in pharmaceutical formulation, particularly for improving the solubility and performance of poorly water-soluble drugs. Hansen solubility parameters (HSPs) provide a systematic approach for estimating solute–solvent affinity before extensive experimental screening is undertaken, and are widely applied in recent studies on deep eutectic and green solvent systems^31^. The concept originates from the Hildebrand solubility parameter, in which the cohesive energy density of a substance is related to a single parameter δ, and substances with similar δ values are expected to be mutually miscible. In the Hansen framework, the total solubility parameter is resolved into three contributions associated with dispersion (δ_d_), polar (δ_p_), and hydrogen-bonding (δ_h_) interactions, such that^32,33^

1 $${\delta ^2}_{t}={\delta ^2}_{d}+{\delta ^2}_{p}+{\delta ^2}_{h}$$

The difference between the solubility parameters of a solvent (*i*) and a solute (*j*) is then expressed as

2 $$\Delta {\delta _{i,j}}=\sqrt {4{{({\delta ^i}_{d} - {\delta ^j}_{d})}^2}+{{({\delta ^i}_{p} - {\delta ^j}_{p})}^2}+({\delta ^i}_{h} - {\delta ^j}_{h}} {)^2}$$

Here Δδ_i, j_ represents the dissimilarity factor, and the superscripts (*i*) and (*j*)correspond respectively to the solvent and the solute.

### Solubility measurement

Solubility was determined using the shake-flask method at temperatures 298.15 ± 0.01 K, 303.15 ± 0.01 K, 308.15 ± 0.01 K, and 313.15 ± 0.01 K under atmospheric pressure (866 ± 2 hPa). The maximum absorbance wavelength (λ_max_) of 4HC was determined as 288 nm through full-spectrum scanning (190–480 nm) in water-ethanol mixed solution. To verify solvent interference prior to analysis, absorbance spectra of mixed solvent (water: ethanol 20:80 v/v) and 100% ADESs were recorded at 190–480 nm. All blanks exhibited A < 0.01 at this wavelength, confirming negligible matrix interference.

Then calibration standards (0–5 × 10^–5^ weight fraction of 4HC) in mixed solvent yielded a linear response (R² = 0.9998). Binary solvent mixtures containing 0–1 weight fraction of ADES were prepared gravimetrically (± 0.1 mg), excess solid 4HC was added, and equilibrated for 72 h in a thermostatic shaker (100 rpm, ± 0.01 K)^34,35^.

At the end of the equilibration period, the samples were centrifuged to remove undissolved solid material, and the resulting supernatant was filtered through a 0.22 μm PTFE membrane to eliminate any remaining particulates. Appropriate aliquots of the filtrate were then diluted with the ethanol–water mixed solvent so that the measured absorbance fell within the validated calibration range and were analyzed by UV–Vis spectrophotometry. The solubility of 4HC was expressed as the mole fraction x₁, calculated from the experimentally determined mass fractions according to Eq. (3)^36^:

3 $$x_{1} = \frac{{\frac{{w_{1} }}{{M_{1} }}}}{{\frac{{w_{1} }}{{M_{1} }} + \frac{{w_{2} }}{{M_{2} }} + \frac{{w_{3} }}{{M_{3} }}}}$$

Where M_i_ and w_i_ denote, respectively, the molar mass and mass fraction of component i. All measurements were performed in triplicate, and mean values were used for further thermodynamic analysis.

### Thermodynamic modeling and solubility correlation

The equilibrium mole fraction solubility of a crystalline solute in a liquid phase is determined by the equality of its chemical potential in the saturated solution and in the pure solid phase, which is conventionally described within the solid–liquid equilibrium (SLE) framework. For a non-electrolyte such as 4-hydroxycoumarin, the general SLE expression may be written as^37^:

4 $$\ln {x_1}= - \ln {\gamma _1}+\frac{{\Delta {_fusH{}}}}{R}(\frac{1}{{{T_{{m_1}}}}} - \frac{1}{T}) - \frac{1}{{RT}}\int_{{{T_{{m_1}}}}}^{T} {\Delta {C_{P1}}dT} +\frac{1}{R}\int_{{{T_{{m_1}}}}}^{T} {\frac{{\Delta {C_{P1}}}}{T}dT}$$

where x_1_ is the mole fraction solubility, R is the gas constant, T and T_m1_ are the system and melting temperatures, Δ_fus_H is the molar enthalpy of fusion, γ_1_ is the activity coefficient of the solute in the saturated solution, and ΔCₚ₁ is the difference in molar heat capacity between the liquid and solid phases.

The short-range contribution is described using local-composition activity-coefficient models, notably the extended NRTL (e-NRTL), Wilson, and UNIQUAC equations, which provide the activity coefficients required in the SLE expressions. This combined treatment affords a rigorous and formally consistent basis for correlating the experimental solubility data of 4HC in ADES-based media and for supporting subsequent formulation design. Notably, UNIQUAC equation parameters are compiled in Table 3, and the mentioned models are fully explained in the Supporting Information (Equations S-1 to S-15).

**Table 3:** The employed components’ UNIQUAC the r and q parameters.

Component	*r*	q
4-hydroxycoumarin	6.1242	4.9160
Water^74^	0.9200	1.4000
ChCl^75^	6.1285	5.1000
Propionic acid^7^	2.8700	2.6120
Acetic acid^76^	2.2024	2.0720
L-(+)-lactic acid^77^	3.6500	3.5000

The quality of the correlation between model predictions and measured solubility was further assessed by means of the average relative deviation percentage (ARD, %), evaluated according to Eq. (5).

5 $$ARD=100(\frac{{\sum\limits_{{i=1}}^{N} {\frac{{\left| {x_{i}^{{\exp }} - x_{i}^{{cal}}} \right|}}{{\left| {x_{i}^{{\exp }}} \right|}}} }}{N})$$

In this equation, $\ln x_{i}^{{\exp }}$ and $\ln x_{i}^{{cal}}$ represent the experimental and calculated mole-fraction solubility and *N* is the total number of experimental data points included in the dataset. Lower ARD values indicate superior agreement and were therefore used as a quantitative criterion to compare the performance of the utilized models for the systems studied.

The model performance, expressed in terms of ARD (%), follows the order: ChCl/Pro (Wilson: 0.34%, e-NRTL: 1.93%, UNIQUAC: 0.93%), ChCl/Ace (Wilson: 0.45%, e-NRTL: 2.12%, UNIQUAC: 0.78%), and ChCl/Lac (Wilson: 0.52%, e-NRTL: 1.87%, UNIQUAC: 0.89%). Notably, the Wilson model demonstrated the best overall performance among the investigated local-composition models, with an average relative deviation of 0.44%.

### The apparent thermodynamic properties of dissolution

The apparent thermodynamic quantities associated with the dissolution of 4-hydroxycoumarin were evaluated within the framework of classical equilibrium thermodynamics. These functions were obtained from relations based on the Gibbs equation and the van’t Hoff formalism, which together allow characterization of the energetic features governing the dissolution process over the investigated temperature range. Calculations were performed at the harmonic mean temperature of the experimental interval, T_hm_ = 305.55 K, corresponding to measurements carried out between 298.15 and 313.15 K^38^.

The standard molar enthalpy of dissolution, ΔH°_soln_, was determined from the temperature dependence of the equilibrium mole-fraction solubility, x_1_, according to^39^

6 $$\Delta H_{{{\mathrm{soln}}}}^{{\mathrm{o}}}= - R{(\frac{{\partial \ln {x_1}}}{{\partial ({\raise0.7ex\hbox{$1$} \!\mathord{\left/ {\vphantom {1 T}}\right.\kern-0pt}\!\lower0.7ex\hbox{$T$}})}})_P}$$

where R is the universal gas constant (8.314 J mol^–1^ K^–1^) and T is the absolute temperature. In practice, ΔH°_soln_ was obtained from the slope of a van’t Hoff plot (ln x_1_ vs. 1/T), a method known as the van’t Hoff plot^40,41^:

7 $$\Delta H_{{{\mathrm{soln}}}}^{{\mathrm{o}}}= - R{(\frac{{\partial \ln {x_1}}}{{\partial ({\raise0.7ex\hbox{$1$} \!\mathord{\left/ {\vphantom {1 T}}\right.\kern-0pt}\!\lower0.7ex\hbox{$T$}} - {\raise0.7ex\hbox{$1$} \!\mathord{\left/ {\vphantom {1 {{T_{hm}}}}}\right.\kern-0pt}\!\lower0.7ex\hbox{${{T_{hm}}}$}})}})_P}$$

Additionally, the other thermodynamic parameter encompassing standard molar Gibbs energy, $\Delta G_{{{\mathrm{soln}}}}^{{\mathrm{o}}}$ and standard molar entropy of dissolution $\Delta S_{{{\mathrm{soln}}}}^{o}$ as been explained and calculated as illustrated in the supporting information section (equations S-15 to S-18).

### Cell culture

The human colorectal adenocarcinoma cell line (HT29) was obtained from the Pasteur Institute of Iran and used as an in vitro model of colon carcinoma. The cells were maintained in Roswell Park Memorial Institute medium (RPMI 1640) supplemented with 10% (v/v) heat inactivated fetal bovine serum, 1% penicillin–streptomycin, and 2 mM·L^-1^ glutamine, all prepared under aseptic conditions in a class II biosafety cabinet^42^. Cultures were incubated at 37 °C in a humidified atmosphere containing 5% CO_2_, and the culture medium was renewed every 2–3 days to preserve cells in the exponential growth phase. Cell morphology, confluence, and integrity were monitored routinely using an inverted phase contrast microscope (Nikon Eclipse 80i, Nikon, Tokyo, Japan), and only monolayers exhibiting typical epithelial morphology and viability greater than 90% (trypan blue exclusion) were employed for cytotoxicity experiments^43^.

### MTT assay

Cytotoxicity of the ADESs–4HC formulations was assessed by the MTT assay, a widely used colorimetric method based on the reduction of the tetrazolium salt MTT [3 (4,5 dimethylthiazol 2 yl) 2,5 diphenyltetrazolium bromide] to insoluble formazan by mitochondrial dehydrogenases in metabolically active cells. The HT29 cells were seeded into 96 well plates at an appropriate density (approximately 5 × 10^3^–1 × 10^4^ cells per well) and allowed to attach for 24 h at 37 °C and 5% CO_2_. At the next step, the serial dilutions of ADESs–4HC (0.3–5 µg·mL^–1^) were prepared in sterile RPMI 1640 medium filtered through a 0.22 μm membrane and added to the wells, followed by a 24 h exposure period under identical incubation conditions^44^.

After treatment, the exposure medium was removed and replaced with 50 µL of MTT solution (2 mg m·L^–1^ in phosphate buffered saline, pH 7.2), and plates were incubated for a further 4 h to permit formation of formazan crystals in viable cells. The supernatant was then carefully aspirated and 150 µL of dimethyl sulfoxide (DMSO) was added to each well; plates were gently shaken in the dark for approximately 1 h to ensure complete dissolution of the formazan.The absorbance was measured at 570 nm with a reference wavelength of 630 nm using a Synergy HT microplate reader (BioTek Instruments Inc., Winooski, VT, USA), and cell viability was calculated as the percentage ratio of the absorbance of treated wells to that of untreated control wells. Eventually, concentration–response curves were fitted by nonlinear regression to a four parameter logistic model to obtain IC_50_ values, defined as the concentration of formulation required to reduce cell viability by 50%, which was used as the principal parameter to compare the cytotoxic potency of the different ADESs–4HC systems^45^.

## Results and discussion

### Hansen solubility parameters (HSP)

Hansen solubility parameters (HSPs) provide a quantitative, three-dimensional framework for describing cohesive interactions in liquids and for predicting the compatibility of a solute with a given solvent or solvent mixture. In this formalism, the total solubility parameter δt is decomposed into dispersive (δ_d_), polar (δ_p_), and hydrogen bonding (δ_h_) components, which together represent the main types of intermolecular forces governing dissolution and miscibility ^46,47^.

By closing the HSPs position of a solvent (or solvent mixture such as an ADESs–drug), the smaller the Hansen distance between them, implying high affinity and a high probability of good solubility; conversely, a large distance typically corresponds to poor miscibility. Recent studies have extended this concept to deep eutectic solvents and natural deep eutectic solvents, illustrating that systematic variation of the acidic component, polarity, and hydrogen bonding capacity of the eutectic mixture shifts its location in Hansen space and can be used to tune solubilizing power in a rational way. For ADESs systems in particular, the δh and δp terms are often dominant because strong, directional hydrogen bonds and dipole–dipole interactions govern the stabilization of polar, hydrogen-bond donor/acceptor solutes such as 4-hydroxycoumarin^48–50^.

**Fig. 1:**
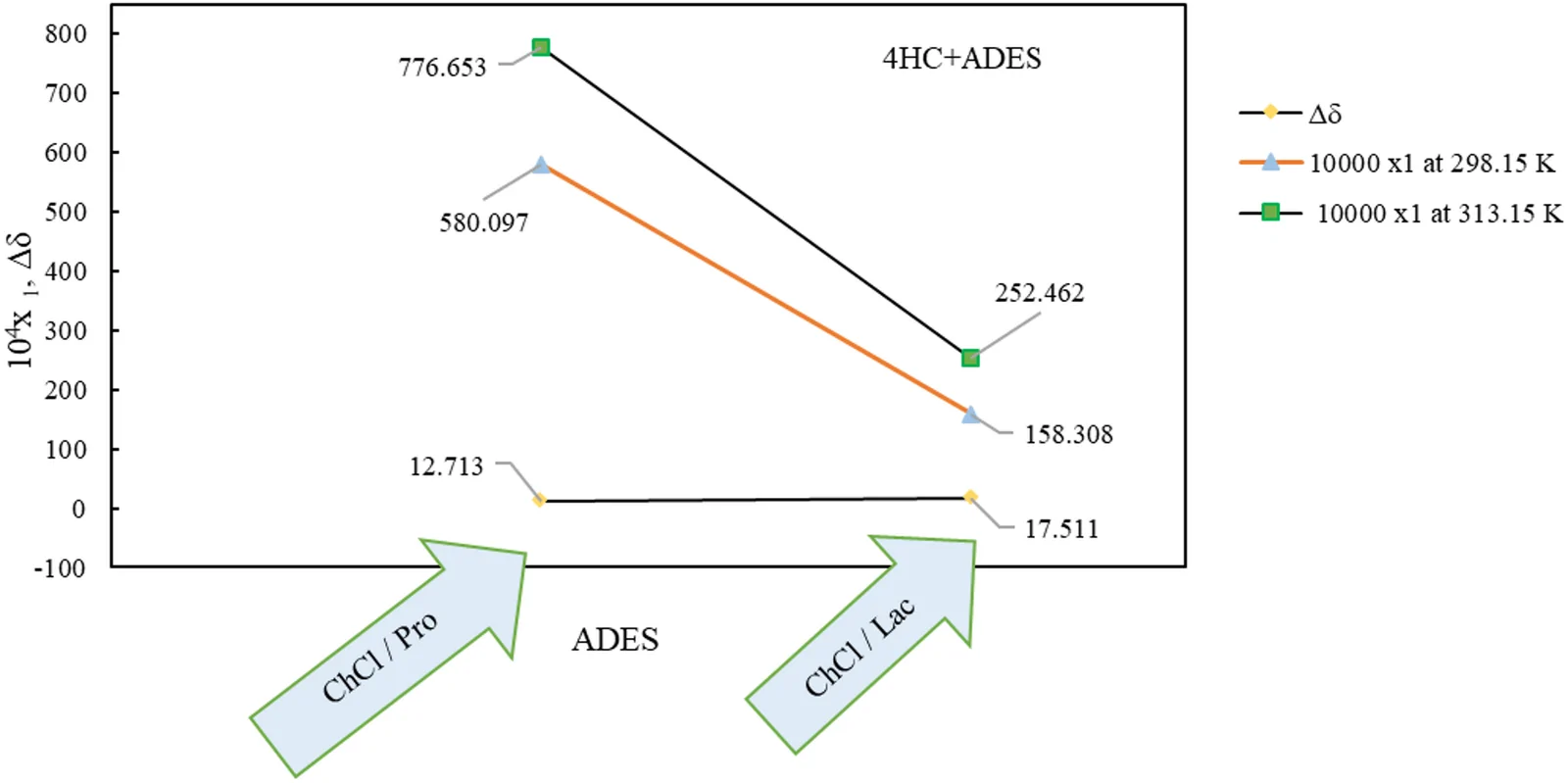
The relationship between mole fraction *x*_1,_ experimental solubility data of 4HC and ∆δ of components in studied systems containing ADES and 4HC. `.

In the context of 4HC, calculating HSPs coordinates for both the solute and a series of ADESs formulations (e.g., choline chloride combined with different organic acids) allows prediction of which compositions are most likely to maximize solute–solvent affinity before performing extensive shake-flask experiments (Table 4; Fig. 1). By correlating the Hansen distance with experimentally measured solubility, it is possible to confirm that systems with lower total HSPs mismatch indeed exhibit higher solubility, thereby validating HSPs-guided screening as an efficient pre-formulation strategy for poorly water-soluble APIs. This approach aligns with current trends in green and rational pharmaceutical design, where computational or semi-empirical tools are integrated with experimental work to minimize trial and error, reduce solvent use, and accelerate the identification of optimal ADESs-based drug-delivery system^51–53^.

**Table 4:** Solubility parameters for ADESs, 4-HC, and the dissimilarity factor (Δδi) between ADESs and 4-HC.

(a) Solubility parameters for the materials.			
Compounds	${\delta _d}$(MPa^1/2^)	${\delta _p}$(MPa^1/2^)	${\delta _h}$(MPa^1/2^)
Water^78^	15.5	16.0	42.3
Choline Chloride^79^	33.2	19.5	18.4
Propionic acid	14.7	5.3	12.4
Acetic acid	14.5	8	13.5
L-(+)-lactic acid	17	8.3	28.4
4-hydroxcy coumarin^80^	20.5	10.5	12.5

(b) $\Delta \delta$ for ADESs and 4HC			
Co-solvents	ChCl/Pro	ChCl/Ace	ChCl/Lac
Solute			
4HC	12.7	12.3	17.5

### Solubility results

**Fig. 2:**
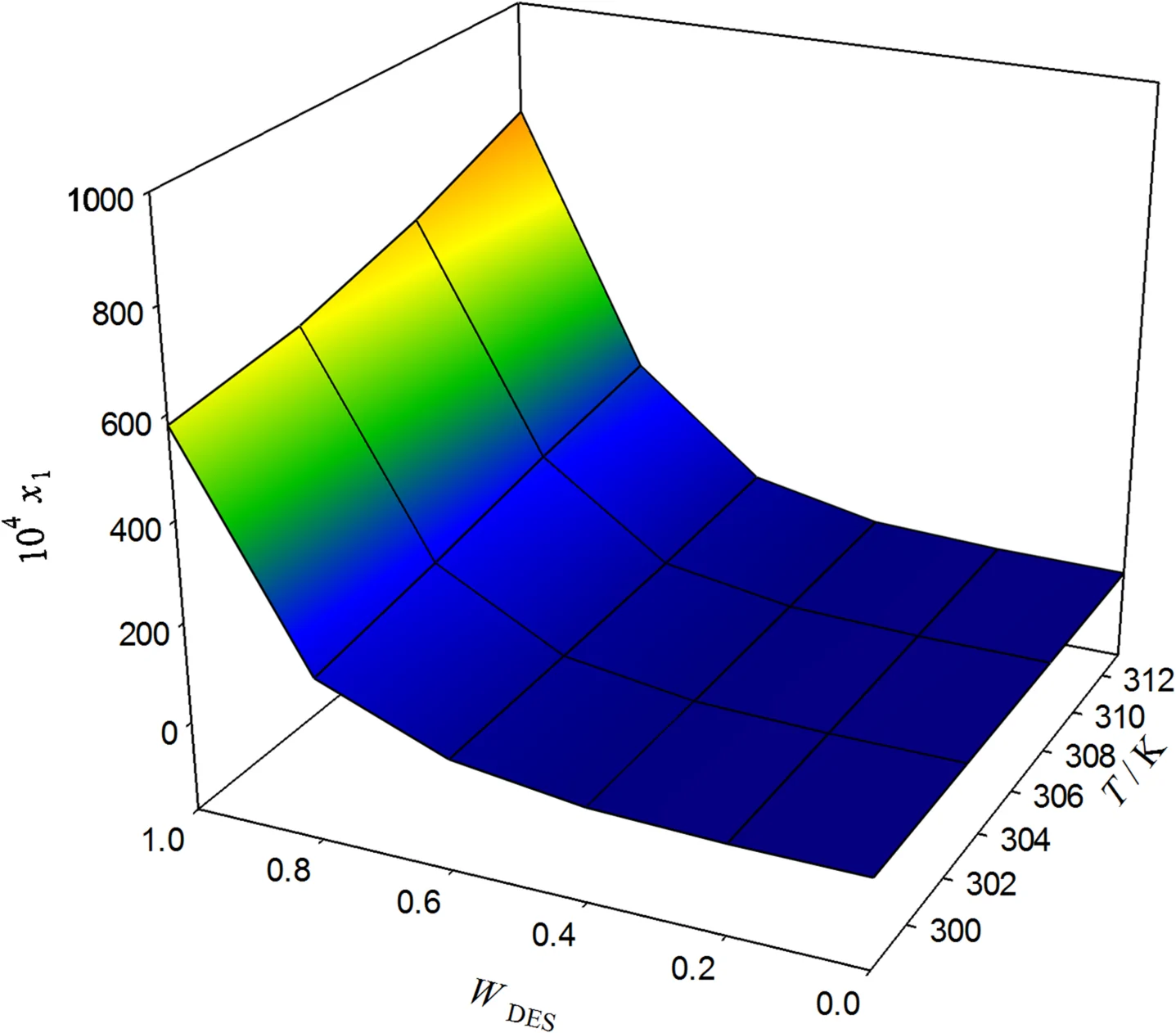
The relationship between solubility of 4HC, mole fraction *x*_*1*_, versus weight fraction of ADES, *w*_*ADES*_, and temperature in aqueous ChCl/Pro solutions.

**Fig. 3:**
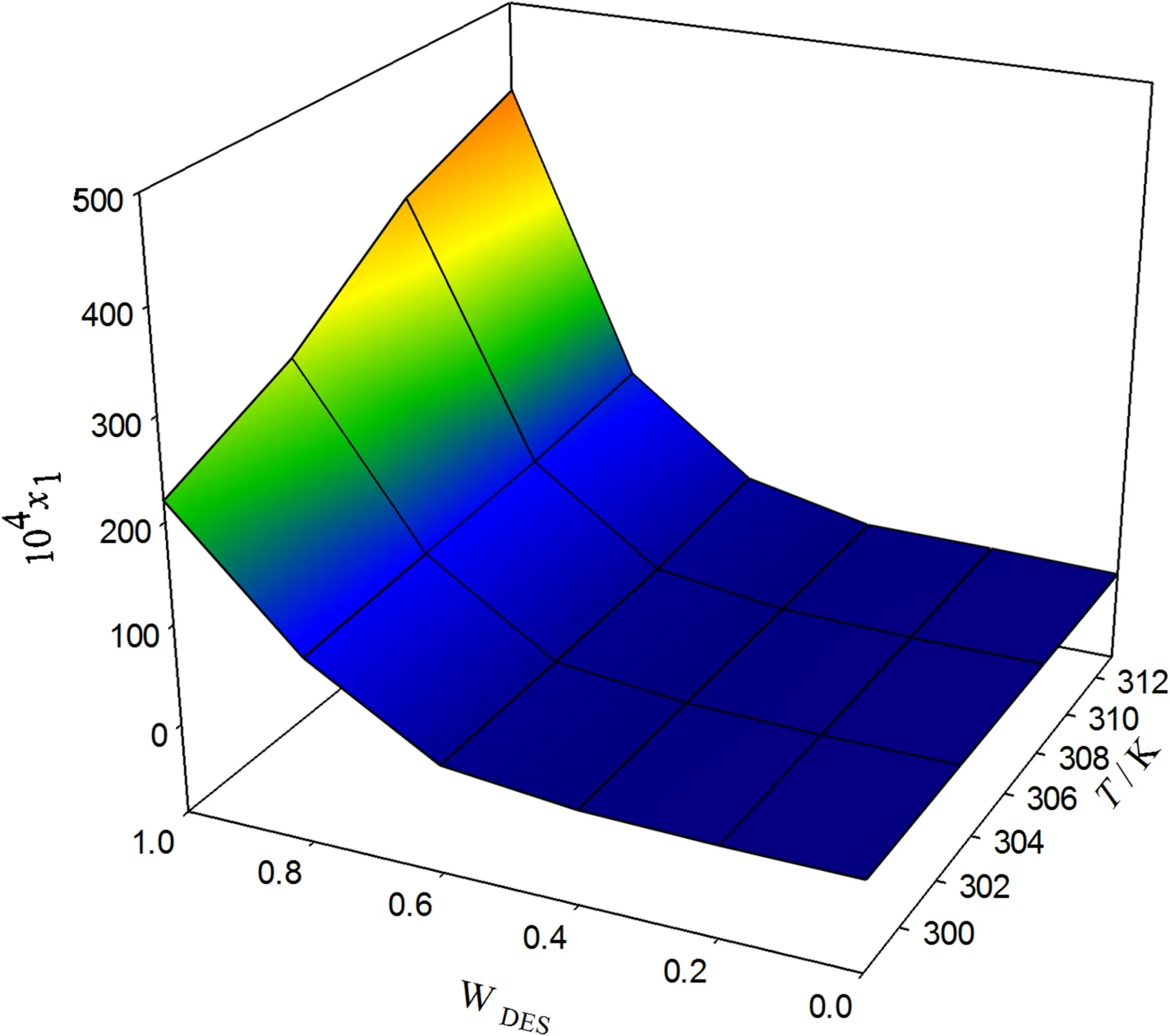
The relationship between solubility of 4HC, mole fraction *x*_*1*_, versus weight fraction of ADES, *w*_*ADES*_, and temperature in aqueous ChCl/Ace solutions.

**Fig. 4:**
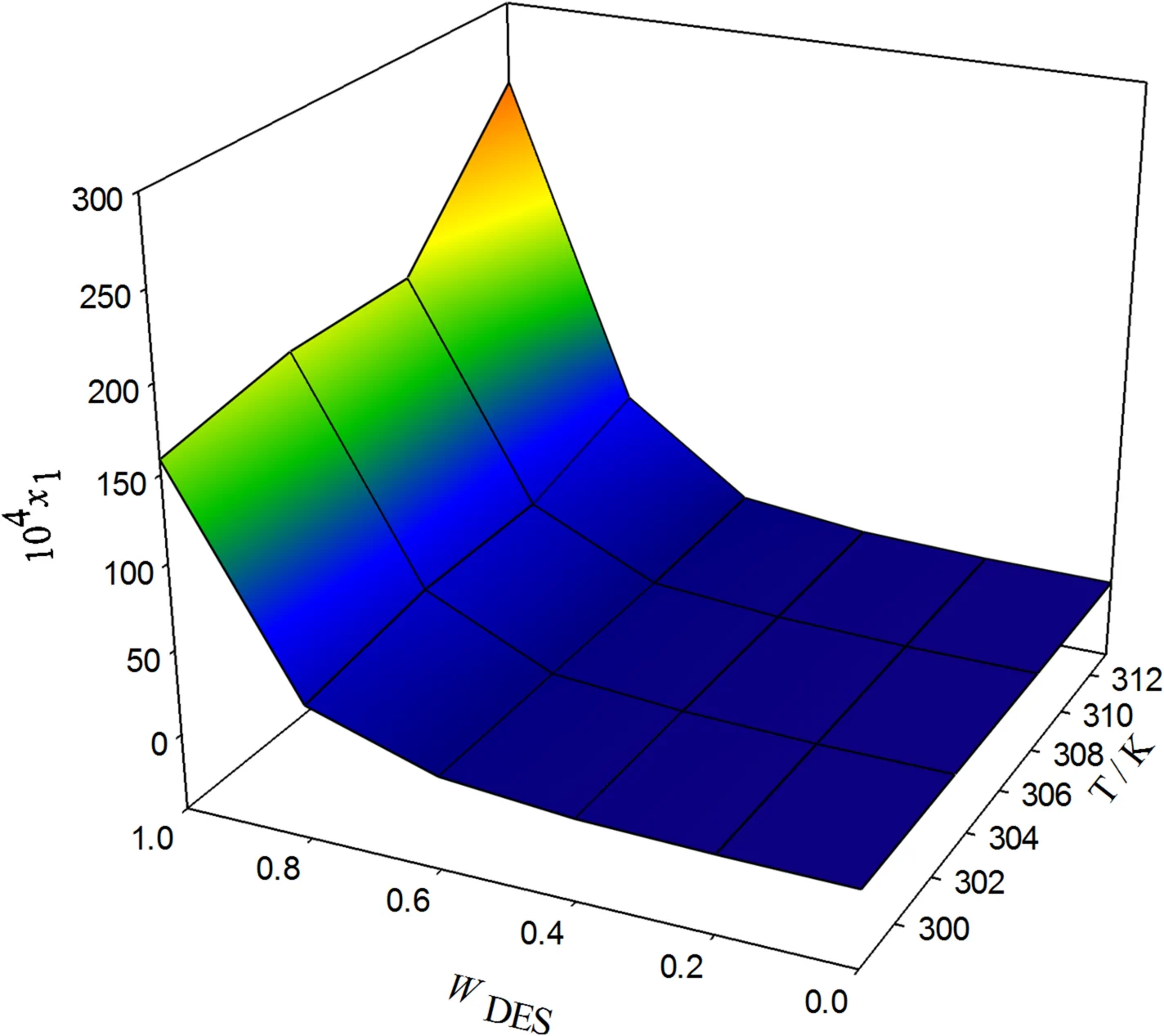
The relationship between solubility of **4HC**, mole fraction *x*_*1*_, versus weight fraction of ADES, *w*_*ADES*_, and temperature in aqueous ChCl/Lac solutions.

The current research assessed the interaction between 4HC and three ADESs is ordered as the ChCl/Pro, ChCl/Ace, or ChCl/Lac under constant conditions of 298.15–313.15 K and 866 hPa which is illustrated in the Table 5; Figs. 2, 3 and 4. The 500-fold enhancement of solubility of this ADESs over water is caused by synergistic molecular interactions. Initially, hydrogen bonding is overwhelmingly dominant which are showing the carbonyl group (C =O) and aromatic ring of 4HC establish strong hydrogen bonds with acidic protons (–COOH) and chloride ions (Cl⁻) in the ADESs, thereby disturbing the crystalline lattice of 4HC. Moreover, the molecular structure of the acidic compounds facilitates solute-solvent interactions. Propionic acid’s longer alkyl chain offers hydrophobic interaction with the coumarin derivatives aromatic system, while acetic acid’s tightness offers tighter packing around the solute. Lactic acid, though possessing hydrogen bonding capability, provides steric hindrance and increased viscosity owing to its secondary hydroxyl group, which restricts dissolution kinetics to some extent.

The observed trend (ChCl/Pro > ChCl/Ace > ChCl/Lac) indicates the effect of minor structural variations in the acid (e.g., alkyl chain length, functional group) on solvent performance. The findings are in accord with established ADESs–drug interaction mechanisms^7,54^.It’s demonstrate that solvent designbalancing hydrogen bonding and hydrophobicitycan enhance experimental solubility. Using these principles, ADESs function as designed solvents to dissolve recalcitrant drugs like 4HC and provide a sustainable alternative to traditional organic solvents^55^.

The solubility enhancement of 4HC in ADESs is controlled by a combination of ion–dipole, dipole–dipole, and hydrogen-bonding (H-bonding) interactions. Specifically, H-bonding between the hydroxyl groups of 4HC and the hydroxyl/chloride moieties of ADESs governs the solvation process. The ADESs possess higher solvating power compared to water due to additional ion–dipole interactions, which supplement H-bonding and dipole–dipole effects^56^. On the other hand, the solubilization efficiency of ADESs is directly connected to their intrinsic intermolecular interactions. For instance, ChCl/Lac, with strong internal interactions, weakens its interaction with 4HC and shows lower solubility improvement, whereas ChCl/Pro, with weaker internal interactions, facilitates stronger ADESs–drug interactions, demonstrating better solubility of 4HC ^57^. This observation highlights the importance of solvent design flexibility in maximizing drug dissolution without requiring energy-intensive processing.

Additionally, the observed solubility ranking perfectly matches the intrinsic acidity strength of the HBD components (pKa values, Table 2), confirming molecular-level hydrogen bonding as the dominant dissolution mechanism.

It is noteworthy that temperature, ADESs composition, and pH interactions significantly control the solubility behavior of 4HC. This investigation identifies the potential of ADESs as environmentally friendly substitutes for conventional organic solvents in drug-delivery, reducing reliance on volatile and toxic solvents. Through the utilization of molecular-level interactions, optimized, energy-efficient drug delivery systems with lowered environmental impact could be engineered^58,59^.

### Modeling of solubility results

The solubility of 4HC in ADESs was correlated using the Wilson, UNIQUAC, and e-NRTL local composition models to elucidate its dissolution behavior in sustainable solvent systems. The derived parameters (Tables 6, 7 and 8) and average relative deviation (ARD%) values (Table 9) revealed that the Wilson model (ARD = 0.34%) outperformed UNIQUAC (0.93%) and e-NRTL (1.93%), particularly for ChCl/Pro-based ADESs, while UNIQUAC showed superior accuracy for ChCl/Ace systems^60^. This compositional dependency underscores the need for tailored model selection in ADESs design. The low ARD% values (< 2%) across all models confirm their reliability for solubility prediction, critical for optimizing green solvent formulations with minimal experimental waste.

**Table 5:** The 4HC experimental and calculated mole fraction respectively (*x*^exp^, *x*^cal^)^a^ in the aqueous ADESs solutions with various weight fractions (*w3*)^b^ within the temperature range *T*/K = 298.15 to 313.15 and pressure (*p* = 866 hPa)^d^ from *e-NRTL*, UNIQUAC and Wilson models.

T / K	e-NRTL model		Wilson model		UNIQUAC model		
	$10^{4} x_{1}^{{\exp }}$	$10^{4} x_{{\mathrm{1}}}^{{{\mathrm{cal}}}}$	$100\frac{{x_{1}^{{\exp }} - x_{{\mathrm{1}}}^{{{\mathrm{cal}}}} }}{{x_{1}^{{\exp }} }}$	$10^{4} x_{{\mathrm{1}}}^{{{\mathrm{cal}}}}$	$100\frac{{x_{1}^{{\exp }} - x_{{\mathrm{1}}}^{{{\mathrm{cal}}}} }}{{x_{1}^{{\exp }} }}$	$10^{4} x_{{\mathrm{1}}}^{{{\mathrm{cal}}}}$	$100\frac{{x_{1}^{{\exp }} - x_{{\mathrm{1}}}^{{{\mathrm{cal}}}} }}{{x_{1}^{{\exp }} }}$
4HC (1) + water (2) + ChCl/Pro (3)							
* w*_*3*_ = 0.0000							
298.15	0.838	0.837	0.12	0.838	0	0.838	0
303.15	1.021	1.021	0	1.021	0	1.021	0
308.15	1.352	1.348	0.3	1.350	0.15	1.352	0
313.15	1.507	1.503	0.27	1.507	0	1.511	-0.27
* w*_*3*_ = 0.2000							
298.15	1.785	1.78	0.28	1.786	-0.06	1.78	0.28
303.15	2.228	2.222	0.27	2.231	-0.13	2.228	0
308.15	2.69	2.696	-0.22	2.706	-0.59	2.703	-0.48
313.15	3.362	3.349	0.39	3.373	-0.33	3.309	1.58
* w*_*3*_ = 0.4000							
298.15	9.941	9.844	0.98	9.929	0.12	10.063	-1.23
303.15	10.325	10.231	0.91	10.249	0.74	10.303	0.21
308.15	11.945	11.619	2.73	11.686	2.17	11.573	3.11
313.15	13.449	13.345	0.77	13.221	1.7	13.617	-1.25
* w*_*3*_ = 0.6000							
298.15	43.895	43.007	2.02	43.934	-0.09	43.147	1.7
303.15	47.062	46.073	2.1	47.445	-0.81	47.437	-0.8
308.15	52.656	52.228	0.81	53.897	-2.36	56.262	-6.85
313.15	65.095	63.726	2.1	66.342	-1.92	67.303	-3.39
* w*_*3*_ = 0.8000							
298.15	146.159	143.135	2.07	146.135	0.02	147.942	-1.22
303.15	185.737	180.771	2.67	185.333	0.22	184.081	0.89
308.15	232.214	222.291	4.27	230.697	0.65	220.282	5.14
313.15	269.178	263.089	2.26	267.743	0.53	256.814	4.59
* w*_*3*_ = 1.0000							
298.15	580.097	580.481	-0.07	580.1	-0.001	578.683	0.24
303.15	612.941	612.795	0.02	613.127	-0.03	614.845	-0.31
308.15	681.501	681.961	-0.07	681.564	-0.01	690.49	-1.32
313.15	776.653	776.429	0.03	776.67	-0.002	788.853	-1.57
4HC (1) + water (2) + ChCl/Ace (3)							
* w*_*3*_ = 0.0000							
298.15	0.838	0.838	0	0.844	-0.72	0.842	-0.48
303.15	1.021	1.021	0	1.024	-0.29	1.021	0
308.15	1.352	1.35	0.15	1.349	0.22	1.352	0
313.15	1.507	1.494	0.86	1.507	0	1.517	-0.67
* w*_*3*_ = 0.2000							
298.15	1.565	1.569	-0.26	1.562	0.19	1.572	-0.45
303.15	1.617	1.644	-1.67	1.623	-0.37	1.616	0.06
308.15	1.695	1.692	0.18	1.704	-0.53	1.695	0
313.15	4.211	3.969	5.75	4.21	0.02	4.167	1.04
* w*_*3*_ = 0.4000							
298.15	4.752	4.527	4.73	4.711	0.86	4.75	0.04
303.15	5.424	5.165	4.78	5.387	0.68	5.423	0.02
308.15	4.977	4.957	0.4	4.914	1.27	4.976	0.02
313.15	5.28	6.383	-20.89	5.282	-0.04	5.291	-0.21
* w*_*3*_ = 0.6000							
298.15	18.184	19.276	-6.01	18.262	-0.43	18.228	-0.24
303.15	19.462	19.85	-1.99	19.557	-0.49	19.46	0.01
308.15	21.972	21.874	0.45	22.255	-1.29	21.975	-0.01
313.15	32.913	26.523	19.41	32.908	0.02	33.101	-0.57
* w*_*3*_ = 0.8000							
298.15	95.004	89.255	6.05	94.882	0.13	94.82	0.19
303.15	104.086	101.277	2.7	104.306	-0.21	104.073	0.01
308.15	112.18	110.67	1.35	111.817	0.32	112.147	0.03
313.15	127.106	132.942	-4.59	127.107	-0.001	126.675	0.34
* w*_*3*_ = 1.0000							
298.15	220.405	222.107	-0.77	220.19	0.1	220.153	0.11
303.15	276.074	276.029	0.02	276.012	0.02	276.039	0.01
308.15	363.458	363.511	-0.01	363.461	-0.001	363.41	0.01
313.15	410.967	411.132	-0.04	410.956	0.003	411.109	-0.03
4HC (1) + water (2) + ChCl/Lac (3)							
* w*_*3*_ = 0.0000							
298.15	0.838	0.831	0.84	0.838	0	0.842	-0.48
303.15	1.021	1.021	0	1.021	0	1.018	0.29
308.15	1.352	1.346	0.44	1.351	0.07	1.35	0.15
313.15	1.507	1.503	0.27	1.507	0	1.508	-0.07
* w*_*3*_ = 0.2000							
298.15	1.59	1.6	-0.63	1.591	-0.06	1.564	1.64
303.15	1.6	1.669	-4.31	1.6	0	1.627	-1.69
308.15	1.538	1.616	-5.07	1.54	-0.13	1.551	-0.85
313.15	2.083	2.101	-0.86	2.083	0	2.075	0.38
* w*_*3*_ = 0.4000							
298.15	3.535	3.565	-0.85	3.535	0	3.602	-1.9
303.15	4.19	3.775	9.9	4.186	0.1	4.065	2.98
308.15	4.259	3.894	8.57	4.237	0.52	4.179	1.88
313.15	4.686	4.541	3.09	4.684	0.04	4.727	-0.87
* w*_*3*_ = 0.6000							
298.15	9.931	9.729	2.03	9.903	0.28	9.962	-0.31
303.15	10.133	10.796	-6.54	10.14	-0.07	10.342	-2.06
308.15	11.77	11.903	-1.13	11.838	-0.58	11.974	-1.73
313.15	12.812	12.94	-1	12.816	-0.03	12.715	0.76
* w*_*3*_ = 0.8000							
298.15	34.196	34.171	0.07	34.257	-0.18	33.64	1.63
303.15	45.463	44.116	2.96	45.461	0.004	45	1.02
308.15	51.552	51.272	0.54	51.48	0.14	50.866	1.33
313.15	65.23	63.794	2.2	65.225	0.01	65.645	-0.64
* w*_*3*_ = 1.0000							
298.15	158.308	158.289	0.01	158.302	0.004	159.4	-0.69
303.15	171.978	171.938	0.02	171.989	-0.01	172.908	-0.54
308.15	202.658	203.318	-0.33	202.651	0.003	204.077	-0.7
313.15	252.462	252.43	0.01	252.454	0.003	251.485	0.39

**Table 6:** The parameters of *e-NRTL *^*a*^ activity coefficient model for the 4HC in the aqueous ADESs solutions.

*T* / K	10^–4^* ∆g*_*wd*_	10^–3^* ∆g*_*dw*_	10^–3^ *∆g*_*Pad*_	10^–4^* ∆g*_*dPa*_	10^–3^ *∆g*_*caw*_	*∆g*_*wca*_	*∆g*_*cad*_	*∆g*_*dca*_	10^–3^* ∆g*_*Paca*_	10^–4^*∆g*_*capa*_	10^–3^*∆g*_*Paw*_	*∆g*_*wPa*_
4HC (1) + water (2) + ChCl/Pro (3)												
298.15	1.83	-3.42	0.10	-5.62	-1.06	-18.95	-12.30	1.74	-1.41	-9.15	-0.02	0.13
303.15	1.62	-1.98	-6.58	-4.40	-0.07	-47.17	-3.06	3.48	-5.49	-8.65	-2.06	0.25
308.15	1.55	-1.53	-3.50	-9.93	0.34	-40.31	-6.03	3.49	-5.59	-151.00	-0.02	0.25
313.15	1.55	-1.23	-9.59	-5.39	0.10	-42.66	-8.83	3.48	-5.55	-80.15	-0.40	0.25

*T* / K	10^–4^*∆g*_*wd*_^*b*^	10^–3^*∆g*_*dw*_	10^–3^ *∆g*_*Acd*_	10^–3^*∆g*_*dAc*_	10^–3^*∆g*_*caw*_	*∆g*_*wca*_	10^–2^ *∆g*_*cad*_	*∆g*_*dca*_	10^–4^* ∆g*_*Acca*_	*∆g*_*caAc*_	10^–4^*∆g*_*Acw*_	*∆g*_*wAc*_
4HC (1) + water (2) + ChCl/Ace (3)												
298.15	1.55	-1.59	0.11	-9.97	1.33	-17.90	-9.14	1.75	-1.41	10.28	-0.02	0.15
303.15	1.39	-0.03	-1.64	-14.46	11.13	134.46	-1.81	5.01	0.45	20.06	7.71	0.35
308.15	1.38	0.03	-2.14	-5.18	2.58	163.25	-12.30	4.08	29.03	-7.77	1.82	0.35
313.15	1.58	-1.49	6.80	-7.66	1.39	-0.88	-14.82	1.75	-1.50	-8.58	16.09	0.13

*T* / K	10^–4^* ∆g*_*wd*_	10^–3^* ∆g*_*dw*_	10^–3^ *∆g*_*Lad*_	10^–3^* ∆g*_*dLa*_	10^–3^* ∆g*_*caw*_	*∆g*_*wca*_	10^–3^* ∆g*_*cad*_	*∆g*_*dca*_	10^–4^*∆g*_*Laca*_	10^–3^*∆g*_*caLa*_	10^–4^*∆g*_*Law*_	*∆g*_*wLa*_
4HC (1) + water (2) + ChCl/Lac (3)												
298.15	1.09	4.00	-2.49	0.01	0.66	158.35	-3.38	4.17	26.37	-0.15	-1.80	0.35
303.15	1.45	-0.58	0.04	-1.16	11.46	-43.27	1.56	3.51	-5.56	-1.90	3.28	0.25
308.15	1.41	-0.32	-0.21	-1.55	0.58	249.83	-0.98	6.07	0.44	-2.12	2.95	0.35
313.15	1.34	0.75	-0.20	-1.99	3.26	-3.30	0.01	2.67	-578.71	-2.68	32.47	0.17

^a^ The non-randomness parameter of the *e-NRTL* model was set equal to 0.3. w = water, d = drug (4HC ), Pa = propionic acid part, ca = cation and anion, Ac = Acetic acid, La = Lactic acid.

**Table 7:** The parameters of Wilson model for the 4HC in the aqueous ADESs solutions.

T / K	10^5^ Λ_dw_	Λ_wd_	10^4^Λ_dPa_	Λ_PAd_	10^4^Λ_dca_	10^3^Λ_cad_	10^4^Λ_wPa_	Λ_Paw_	10^4^Λ_wca_	10^3^Λ_caw_	10^3^Λ_Paca_	Λ_caPa_
4HC (1) + water (2) + ChCl/Pro (3)												
298.15	0.58	3.85	5.19	6.75	-0.22	-0.28	9.79	5.29	11.87	94.30	3.32	15.20
303.15	0.89	3.68	8.11	6.57	10.45	-0.18	4.86	4.43	54.41	41.10	10.31	-159.90
308.15	9.86	3.40	23.28	5.82	-0.23	0.01	0.26	4.51	3.06	329.70	0.83	-10.00
313.15	-8.53	2.91	-1.23	4.99	-1.71	0.36	2.66	4.36	3.06	-1.00	0.83	-7.37

*T* / K	10^5^ *Λ*_*dw*_	*Λ*_*wd*_	10^4^*Λ*_*dAc*_	*Λ*_*Acd*_	10^4^*Λ*_*dca*_	10^3^*Λ*_*cad*_	10^4^*Λ*_*wAc*_	*Λ*_*Acw*_	10^3^*Λ*_*wca*_	10^3^*Λ*_*caw*_	10^3^*Λ*_*Paca*_	*Λ*_*caAc*_
4HC (1) + water (2) + ChCl/Ace (3)												
298.15	0.24	3.86	5.19	4.83	-0.22	-282.10	9.79	5.20	11.87	94.70	3.32	15.20
303.15	-0.76	3.69	8.55	4.75	10.82	-158.00	48.60	5.22	54.42	48.00	13.14	-163.10
308.15	6.53	3.46	3.43	4.45	-23.42	-0.13	2.57	7.26	3.06	326.60	0.83	-10.00
313.15	-8.22	2.96	-1.03	3.83	-1.52	338.90	2.66	6.86	3.06	-1.34	0.83	-7.40

*T* / K	10^5^ *Λ*_*dw*_	Λ_wd_	10^4^*Λ*_*dLa*_	*Λ*_*Lad*_	10^4^*Λ*_*dca*_	10^3^*Λ*_*cad*_	10^4^*Λ*_*wLa*_	*Λ*_*Law*_	10^3^*Λ*_*wca*_	10^3^*Λ*_*caw*_	10^3^*Λ*_*Paca*_	*Λ*_*caAc*_
4HC (1) + water (2) + ChCl/Lac (3)												
298.15	-0.24	3.85	6.32	4.45	-0.21	-154.70	9.80	10.43	11.87	0.16	3.32	0.02
303.15	-0.93	3.68	7.93	4.29	10.32	-95.80	48.70	8.52	54.48	0.07	13.13	-0.17
308.15	3.36	3.51	0.47	3.99	-0.25	-0.13	2.57	12.69	3.06	0.32	0.83	-0.01
313.15	-2.26	3.36	-9.93	3.99	-8.39	-54.30	2.66	8.56	3.06	0.00	0.83	-0.01

w = water, d = drug (4-hydroxycoumarin), Pa = propionic acid part, ca. = cation and anion, Ac = Acetic acid, La = Lactic acid.

**Table 8:** The parameters of UNIQUAC model for the 4HC in the aqueous ADESs solutions.

T / K	10^-4^ ∆u_wd_	10^-3^ ∆u_dw_	10^-3^ ∆u_Pad_	10^-3^ ∆_udPa_	∆u_caw_	10^-3^ ∆u_wca_	10^-3^ ∆u_cad_	∆u_dca_	∆uPa_ca._	∆u_capa_	10^-3^ ∆u_Paw_	10^-3^ ∆u_wPa_
4HC (1) + water (2) + ChCl/Pro (3)												
298.15	-13.38	2.38	0.05	0.33	-1.52	-37.25	0.01	-2.29	-2.51	2.11	-9.72	726.89
303.15	-5.53	2.36	1.95	3.10	-1.52	-45.64	0.01	-2.48	-0.28	2.22	-7.00	154.68
308.15	-164.36	2.65	1.57	3.98	-0.38	-17.34	2.84	-2.77	22.16	1.00	-7.01	-0.68
313.15	234.89	1.80	5.69	7.95	-1.52	-34.67	3.94	-3.15	38.42	2.00	-5.37	-1.31

*T* / K	10^-3^ *∆u*_*wd*_	10^-3^ *∆u*_*dw*_	10^-3^ *∆u*_*Acd*_	10^-3^ *∆u*_*dAc*_	*∆u*_*caw*_	10^-3^ *∆u*_*wca*_	10^-3^ *∆u*_*cad*_	*∆u*_*dca*_	*∆uAc*_*ca.*_	*10*^*-3*^ *∆u*_*caAc*_	*10*^*-3*^ *∆u*_*Acw*_	*10*^*-4*^ *∆u*_*wAc*_
4HC (1) + water (2) + ChCl/Ace (3)												
298.15	1.92	-4.90	1.36	-3.97	-0.61	-0.61	1.51	1.60	-1.89	2.73	1.07	-4.41
303.15	1.57	-4.64	1.24	-4.04	-0.61	-1.28	1.37	1.60	-1.89	2.73	3.33	-4.41
308.15	2.38	-5.02	1.21	-4.18	-0.62	-0.60	1.32	1.60	-1.89	2.73	0.46	-4.41
313.15	1.14	-4.04	1.03	-4.24	2.68	-1.57	1.08	1.59	-1.88	2.73	29.53	-4.41

*T* / K	10^-3^ *∆u*_*wd*_	10^-3^ *∆u*_*dw*_	10^-3^ *∆u*_*Lad*_	10^-3^ *∆u*_*dLa*_	*∆u*_*caw*_	*∆u*_*wca*_	10^-3^ *∆u*_*cad*_	*∆u*_*dca*_	*∆u*_*Laca*_	*10*^*-3*^ *∆u*_*caLa*_	*10*^*-3*^ *∆u*_*Law*_	*10*^*-3*^ *∆u*_*wLa*_
4HC (1) + water (2) + ChCl/Lac (3)												
298.15	0.25	-0.80	3.37	0.01	-1.52	0.03	0.00	-623.59	19.68	0.04	-2.38	-5.03
303.15	1.13	-3.98	9.38	-8.09	-1.52	2.18	11.75	-6.09	6.58	-1.55	-4.21	3.58
308.15	1.13	-4.01	9.29	-8.32	-1.52	2.69	11.61	-6.06	6.57	-1.74	-5.04	4.37
313.15	1.21	-4.16	9.63	-8.59	-1.52	3.20	12.22	-6.07	6.57	-1.92	-5.38	4.38

The study highlights ADESs as environmentally benign alternatives for pharmaceutical applications, leveraging their low toxicity, biodegradability, and tunability. The Wilson model’s precision, for instance, reduces iterative testing, aligning with energy-efficient process design^61^.

**Table 9:** The calculated average relative deviation percent (*ARD%*) for the solubility of the 4HC in the aqueous ADESs solutions at the temperature range ^a^ *T*/K = 298.15 to 313.15 and pressure (^b^*p* = 866 hPa) from different models.

		*ARD%*	
*T* / K	*e-NRTL*	Wilson	UNIQUAC
4HC (1) + water (2) + ChCl/Pro (3)			
298.15	0.92	0.05	0.78
303.15	1.00	0.33	0.37
308.15	1.40	0.99	2.82
313.15	0.97	0.75	2.11
Average	1.07	0.53	1.52
4HC (1) + water (2) + ChCl/Ace (3)			
298.15	2.98	0.40	0.25
303.15	1.86	0.34	0.02
308.15	0.41	0.61	0.2
313.15	8.60	0.2	0.48
Average	3.46	0.39	0.24
4HC (1) + water (2) + ChCl/Lac (3)			
298.15	0.74	0.09	1.10
303.15	0.40	0.04	1.44
308.15	2.67	0.24	1.11
313.15	1.24	0.01	0.52
Average	1.26	0.10	1.04

ARD% = Percent of average relative deviation.

^*a*^ Standard uncertainty u(*T*) = 0.01 K.

^b^ Standard uncertainty u*(P)* = 10 hPa .

### Thermodynamic properties of dissolution

**Fig. 5:**
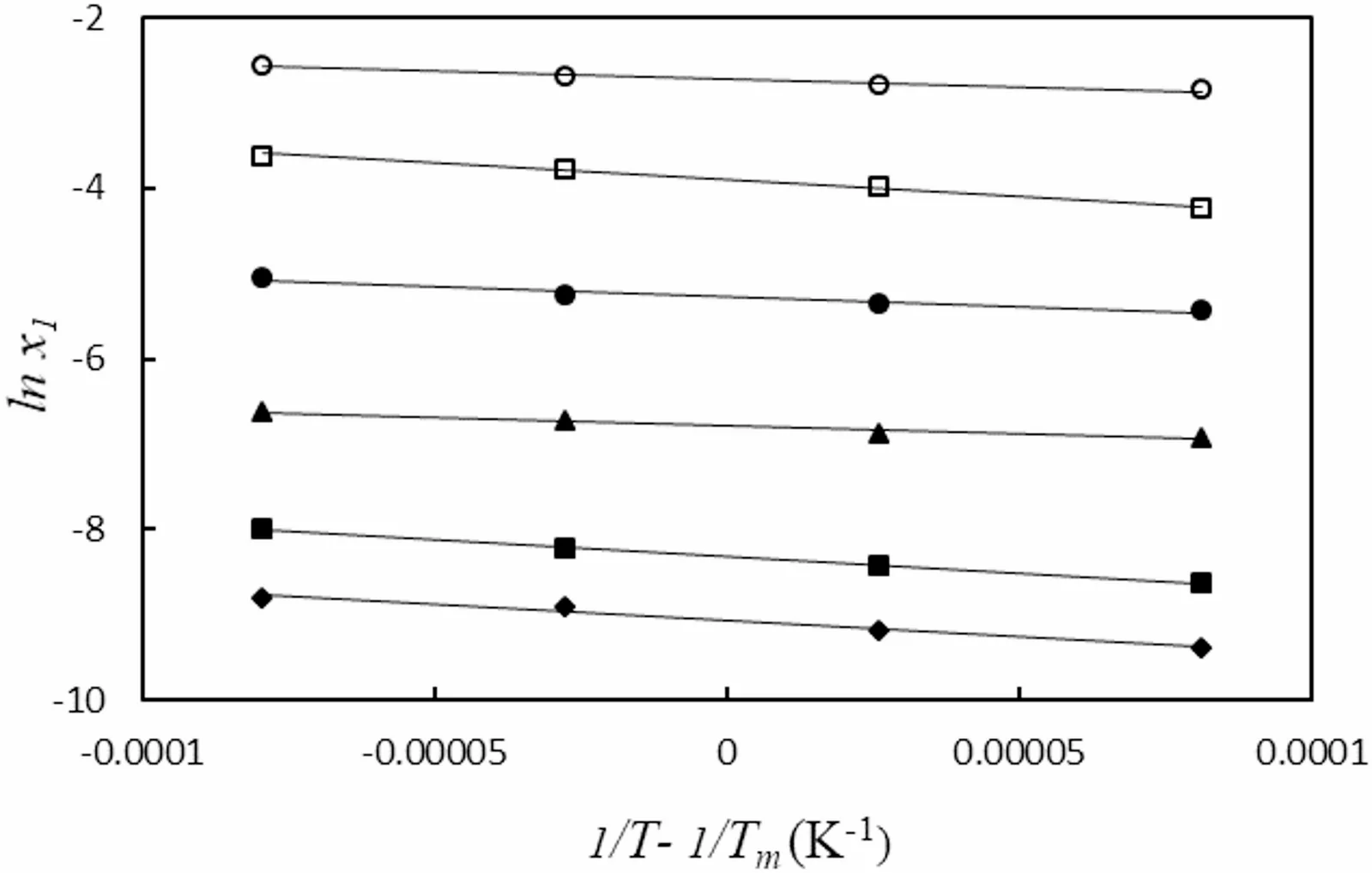
Plot of *lnx1* vs. (*1/T- 1/Tm*); in aqueous ChCl/Pro solutions at different weight fraction of ADES (*w*_*ADES*_): 0.0000(filled diamond), 0.2000 (filled square), 0.4000 (filled triangle), 0.6000 (filled circle), 0.8000 (unfilled square), 1.0000 (unfilled circle).

**Fig. 6:**
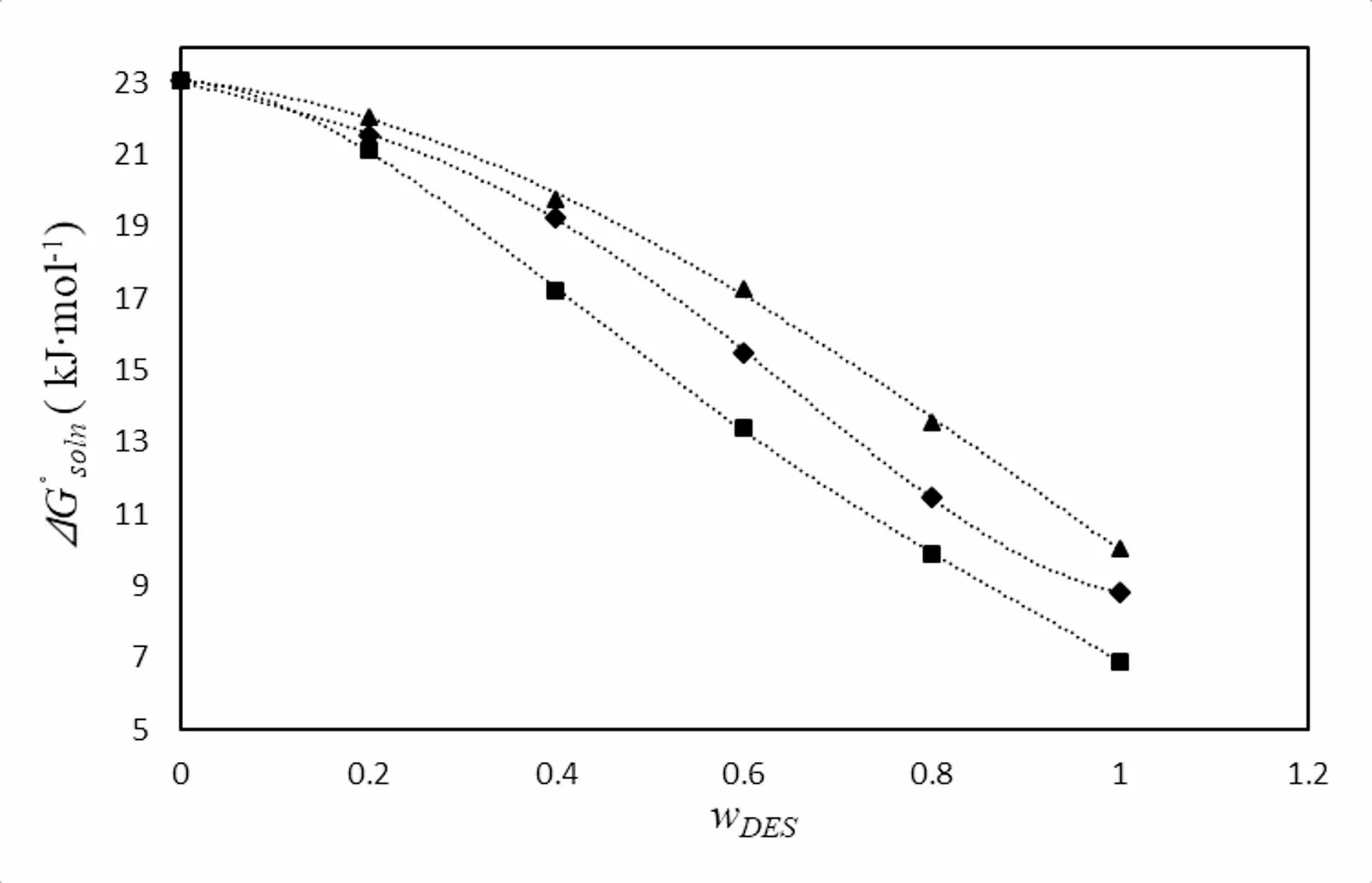
The Δ*G* ° values relative to dissolution process of 4HC in the aqueous ADES solutions at 305.548 K, ChCl/Pro (filled square), ChCl/Ace (filled diamond), ChCl/Lac (filled triangle).

The plots of solubility data, $$\ln x_{1}$$ versus $$\left( {{\raise0.7ex\hbox{$1$} \!\mathord{\left/ {\vphantom {1 T}}\right.\kern-\nulldelimiterspace} \!\lower0.7ex\hbox{$T$}} - {\raise0.7ex\hbox{$1$} \!\mathord{\left/ {\vphantom {1 {T_{{hm}} }}}\right.\kern-\nulldelimiterspace} \!\lower0.7ex\hbox{${T_{{hm}} }$}}} \right)$$for 4HC in aqueous ADESs solutions containing ChCl/Pro were represented in Fig. 5 to evaluate the thermodynamic characteristics of dissolution. Moreover, the results of ($$\Delta H_{{so\ln }}^{o}$$, $$T_{m} \Delta S_{{so\ln }}^{o}$$ and $$\Delta G_{{so\ln }}^{o}$$) are collected in Table 10. Due to the positive values of the standard molar Gibbs energy and dissolution enthalpy $$\Delta G_{{so\ln }}^{o}$$ and $$\Delta H_{{so\ln }}^{o}$$in all systems, the processes of 4HC dissolution in aqueous ADESs solutions are always endothermic. The values decline as the weight fraction of ADESs increases, illustrating that the solubility of 4HC in these types of solvents increases as the ($$\Delta G_{{so\ln }}^{o}$$) values decrease (Fig. 6). On the other hand, $${T_{hm}}\Delta S_{{so\ln }}^{o}$$ is positive in all the solutions investigated in this study, and its values as Δ *Sº* are lower than those of $$\Delta H_{{so\ln }}^{o}$$. The table 10 depicts the computed (*ξ*_*H*_) and (*ξ*_*TS*_) values. Based on the mentioned data, enthalpy is the major contribution to the standard molar Gibbs energy (Δ*G*οsoln) of the dissolution process of 4HC.

**Table 10:** Thermodynamic functions for dissolution process at different weight fractions of ADESs (*w3*)^a^ at mean temperature^b^ and pressure (^c^*p* = 866 hPa).

w_3_	$\Delta H_{{so\ln }}^{^\circ }$/ kJ mol^-1^	$T_{M} \Delta S_{{so\ln }}^{^\circ }$/ kJ mol^-1^	$\Delta G_{{so\ln }}^{^\circ }$/ kJ mol^-1^	$\xi _{H}$	$\xi _{{TS}}$
4HC (1) + water (2) + ChCl/Pro (3)					
0.0000	31.73	8.69	23.04	78.51	21.49
0.2000	32.41	11.28	21.12	74.17	25.83
0.4000	16.3	-0.94	17.23	94.57	5.43
0.6000	20.02	6.64	13.38	75.09	24.91
0.8000	31.95	22.05	9.9	59.17	40.83
1.0000	15.2	8.29	6.91	64.71	35.29
4HC (1) + water (2) + ChCl/Ace (3)					
0.0000	31.73	8.69	23.04	78.51	21.49
0.2000	46.38	24.82	21.56	65.14	34.86
0.4000	3.61	-15.65	19.26	32.6	67.4
0.6000	29.34	13.85	15.49	67.93	32.07
0.8000	14.7	3.22	11.48	82.03	17.97
1.0000	33.33	24.5	8.83	57.64	42.36
4HC (1) + water (2) + ChCl/Lac (3)					
0.0000	31.73	8.69	23.04	78.51	21.49
0.2000	11.81	-10.25	22.06	53.54	46.46
0.4000	13.42	-6.37	19.78	67.82	32.18
0.6000	14.15	-3.13	17.28	81.89	18.11
0.8000	32.05	18.48	13.57	63.43	36.57
1.0000	24.21	14.19	10.03	63.05	36.95

### The cytotoxicity effect of ADESs-4HC

**Fig. 7:**
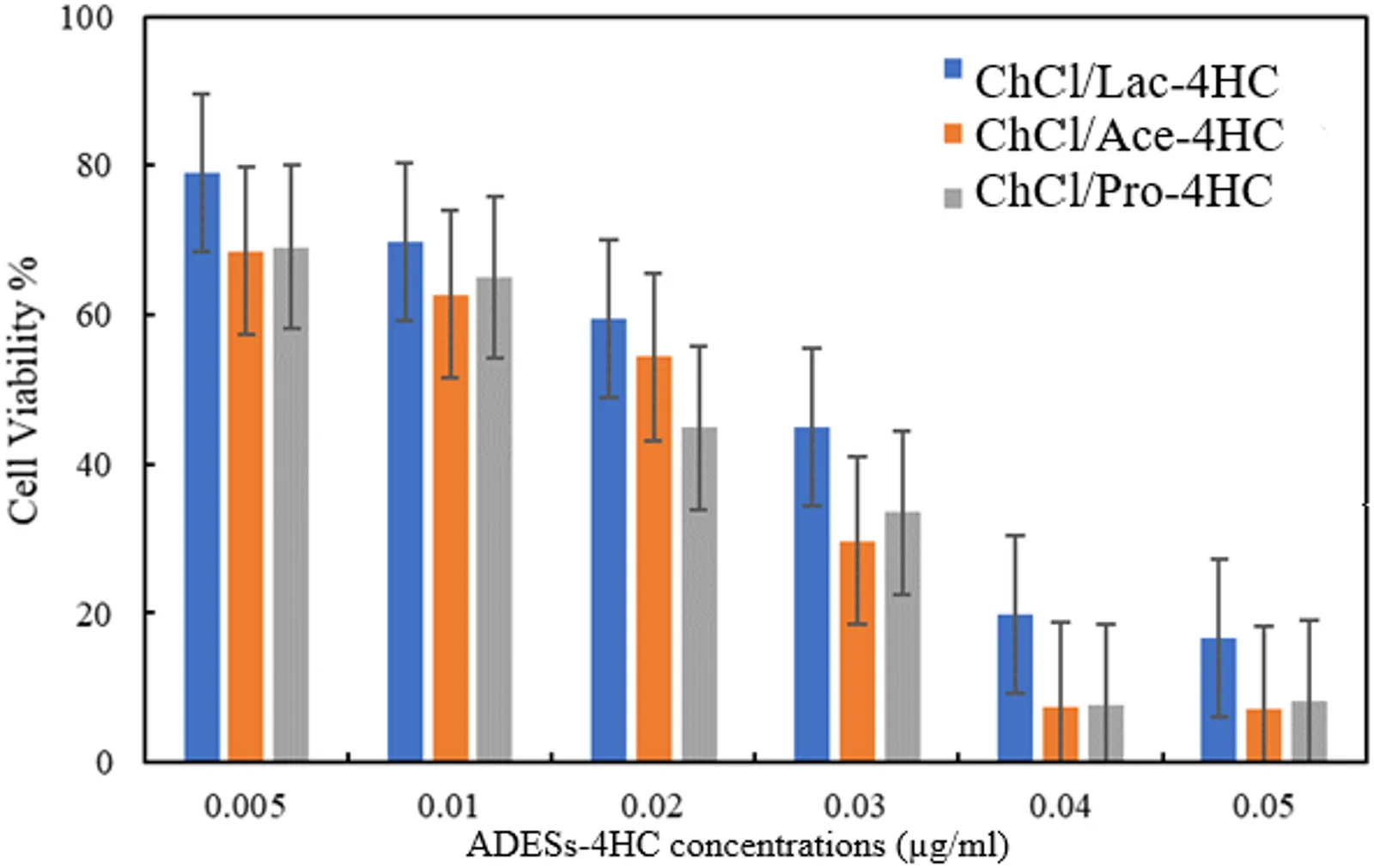
Viability of the human colon adenocarcinoma cell line HT29 dose-response curves of the mentioned ADESs-4HC concentrations (µg/ml).

The cytotoxicity of 4HC-functionalized acid-based deep eutectic solvent (ADES) analogs prepared in situ was determined against HT29 cancer cells using the MTT assay. The IC_50_ values revealed a unique trend of cytotoxicity: ChCl/Ace-4HC > ChCl/Pro-4HC > ChCl/Lac-4HC (Fig. 7), with all exhibiting moderate activity (IC_50_ = 20–100 µg·mL^-1^). This trend underscores the critical role of acidic functional groups (acetate, propionate, and lactate) in modulating bioactivity.

Accordingly, the cytotoxic potential of the ADESs-4HC was differentiated into the following levels: very active, IC_50_ ≤ 20 µgmL^-1^; moderately active, IC_50_ > 20–100 µgmL^-1^; weakly active, IC_50_ > 100–1000 µg·mL^-1^; and inactive, IC_50_ > 1000 µg/mL. Under these experimental conditions, analysis showed the highest cytotoxic effect of the compounds on the HT29 cell line to be in the moderately active range^62^. On the contrary, the reduced activity of propionate and lactate derivatives may be indicative of steric hindrance or utilization of secondary metabolic pathways^63^.

To comparison of the solubility enhancement and Cytotoxicity with literature, ChCl/Pro achieved 500-fold improvement vs. water, surpassing ChCl/oxalic acid/ibuprofen (150-fold)^64^, ChCl/levulinic acid/naproxen (320-fold)^65^, and approaching ChCl/glycerol/curcumin (450-fold)^66^. This confirms ADESs as superior green solubilizers for poorly soluble drugs (Table S-1).

On the other hand the cytotoxicity comparison IC_50_ values (25–85 µg·mL^-1^, HT29) indicate moderate bioactivity comparable to literature DESs-drug systems (18–35 µg·mL^-1^, MCF7/HeLa)^67–69^. While therapeutically relevant, further structural optimization is required for clinical translation (Table S-2).

The ionic nature of ADESs also influences their cytotoxicity, conceivably through interactions with intracellular proteins or acetyl-CoA metabolism (acetate/propionate) and redox homeostasis (lactate) modulation. Such mechanistic insights are consonant with sustainable chemistry goals, as tunable ADESs could offer safer analogs of classical toxic solvents for pharmaceutical applications. Importantly, the reported moderate cytotoxicity suggests a balance between bioactivity and biocompatibility, a critical aspect of green drug design. In addition to therapeutic use, this work underscores the overall utility of ADESs in sustainably prepared media, e.g., as reaction media or biodegradable carriers. To ensure compatibility with green chemistry principles, future studies must explore molecular mechanisms (e.g., apoptosis induction) and eco-toxicity profiles^70,71^. Coupling cytotoxicity information with sustainability measures, this work aids in the development of functional, environmentally benign solvents for biomedical and industrial applications^72,73^.

### Implications for pharmaceutical formulation and drug-delivery

The pronounced solubility enhancement of 4HC in choline chloride-based ADESs, particularly in the ChCl/Pro system, has direct implications for pharmaceutical formulation. The 500-fold increase in solubility relative to water suggests that ADESs-based vehicles could substantially reduce the required dose or eliminate the need for conventional organic co-solvents in liquid and semi-solid dosage forms. Hansen solubility parameter analysis further indicates that hydrogen-bonding and acidity-driven affinity can be used as rational design criteria when selecting or tailoring ADESs compositions for coumarin scaffolds. From a safety standpoint, the HT29 MTT data show moderate cytotoxicity in the 20–100 µg/mL range, which, together with the composition-dependent effects, provides an initial window for acceptable exposure levels in future in vivo or ex vivo studies. Overall, these findings position choline chloride-based acidic deep eutectic solvents, and in particular ChCl/Pro, as promising solubilizing excipients for green 4HC formulations and potential cancer-oriented drug-delivery systems.

## Conclusions

This study systematically investigated the solubility of 4HC in three ADESs—ChCl/Pro, ChCl/Ace, and ChCl/Lac—over a defined temperature range and a series of solvent compositions. The experimental data demonstrated a substantial enhancement of 4HC solubility relative to pure water, with the solubilizing ability of the systems following the order ChCl/Pro > ChCl/Ace > ChCl/Lac and increasing with both temperature and ADESs mass fraction.

Hansen solubility parameters (HSPs) analysis provided a consistent theoretical framework for interpreting these trends, confirming that ADESs formulations exhibiting the smallest Hansen distance from 4HC, particularly ChCl/Pro, afford the highest solute–solvent affinity and the greatest solubility enhancement. Thermodynamic treatment based on van’t Hoff relationships indicated that dissolution of 4HC in the studied ADESs media is endothermic and predominantly enthalpy-driven, reflecting the formation of favorable specific interactions such as hydrogen bonding and ion–dipole contacts. Local-composition activity-coefficient models (Wilson, UNIQUAC, and e-NRTL) reproduced the experimental solubility data with low deviations, supporting their applicability for predictive modeling in electrolyte-containing green solvent systems.

Cytotoxicity assays using HT29 human colorectal adenocarcinoma cells showed moderate antiproliferative effects of the ADES–4HC formulations, with potency ranked as ChCl/Ace–4HC > ChCl/Pro–4HC > ChCl/Lac–4HC, indicating a clear dependence on the nature of the acidic component. These observations are consistent with current evidence that choline-based DESs and ADESs display composition- and concentration-dependent biological responses, influenced by differences in hydrogen-bond donor structure and associated effects on cellular metabolism and redox balance.

Overall, the findings confirm that acidic deep eutectic solvents constitute versatile, tunable, and comparatively benign media capable of markedly improving the solubility of poorly water-soluble drugs such as 4HC while maintaining an acceptable cytotoxicity profile in vitro investigations. The integration of experimental solubility measurements, thermodynamic modeling, Hansen-parameter analysis, and cytotoxicity evaluation provides a coherent, academically rigorous basis for the rational design of ADESs-based pharmaceutical formulations within the broader context of green chemistry and sustainable process development. Furthermore, beyond fundamental solution thermodynamics, the present work provides a preformulation framework for exploiting acidic deep eutectic solvents as solubilizing excipients for 4-hydroxycoumarin, thereby supporting the future development of green and pharmaceutically relevant dosage forms.

## Supplementary Information

Below is the link to the electronic supplementary material.


Supplementary Material 1


## Data Availability

The datasets generated and analyzed during the current study are available from the corresponding author on reasonable request.
