# Supplementary material for: Molecular interactions, solubility, and cytotoxic activity of 4‑hydroxycoumarin in choline chloride‑based acidic deep eutectic solvents
Source: Sci Rep. 2026 Jun 15;16:27329. doi: 10.1038/s41598-026-56725-w (PMC13530267; doi:10.1038/s41598-026-56725-w)
Supplement: Supplementary file 1 [file 41598_2026_56725_MOESM1_ESM.docx]

**Molecular Interactions, Solubility, and Cytotoxic Activity of 4‑Hydroxycoumarin in Choline Chloride‑Based Acidic Deep Eutectic Solvents**

**Mohammad Khorsandi****^1,2^, Hemayat Shekaari**^[1]^**, ^1^, Masoumeh Mokhtarpour^1^, Parisa Akbarzadeh Gondoghdi^1^, Hamed Hamishehkar^2^**

^1^Department of Physical Chemistry, University of Tabriz, Tabriz, Iran

^2^ Drug Applied Research Center, Tabriz University of Medical Sciences, Tabriz, Iran

**Thermodynamic modeling and solubility correlation**

Thermodynamic modeling is an essential tool in contemporary pharmaceutical science for interpreting and predicting the solubility of active pharmaceutical ingredients and for rational solvent selection in complex fluid media. In many practical applications, the heat‑capacity contribution is small or only weakly temperature‑dependent over the experimental range and can therefore be neglected, leading to the simplified expression [1];

| $\ln x_{1}=\frac{\Delta_{fus}H}{R}(\frac{1}{T}_{m}-\frac{1}{T})-\ln\gamma_{1}$ | (S-1) |
| --- | --- |

which is widely employed for correlating the temperature dependence of solubility and for extracting thermodynamic parameters from experimental data. Within this framework, accurate values of the melting temperature, heat of fusion, and activity coefficient are essential for a reliable description of the solubility behaviour of 4HC in aqueous and ADES‑containing solution.

For multicomponent electrolyte solutions such as aqueous acidic deep eutectic solvent systems, the molar excess Gibbs energy in the asymmetric convention, *G*^ex∗^, is commonly partitioned into long‑range and short‑range contributions according to:

| $\frac{G^{ex*}}{RT}=\frac{G^{ex*,LR}}{RT}+\frac{G^{ex*,SR}}{RT}$ | (S-2) |
| --- | --- |

where, the superscript “LR” denotes long‑range electrostatic interactions, typically represented by an extended Pitzer–Debye–Hückel term, while “SR” refers to specific, short‑range interactions such as hydrogen bonding and local composition effects.

**Wilson model**

Established in the Wilson model mostly by composition and temperature, the activity coefficients are as follows :

| $\ln\gamma_{i}=1-\ln(\sum_{j=1}^{n} (\Lambda_{ij}x_{j})-\sum_{k=1}^{n} (\frac{\Lambda_{ki}x_{k}}{\sum_{j=1}^{n} (\Lambda_{kj}x_{j})}))$ | (S-3) |
| --- | --- |

where Λ *ij* is demonstrative of the binary interaction parameter based on characteristic energy (*λ*) and solute	and	solvent molar	volumes (ν), which could be calculated using	Eq. S-4:

⎟

|  | (S-4) |
| --- | --- |

***e-NRTL* model**

The *e-NRTL* model is thoroughly documented in reference [2]:

The activity coefficient for each species is calculated by integrating the Pitzer–Debye–Hückel term with the NRTL term. This method facilitates the calculation of (γ₁) via Equations S-5and S-6:

| ln(*γ* * ) = ln(*γ* **PDH* ) + ln(*γ* **NRTL* ) | (S-5) |
| --- | --- |
| $\ln\gamma_{i}=\frac{\sum_{j=1}^{m} \tau_{ij}G_{ij}x_{j}}{\sum_{i=1}^{m} G_{li}x_{l}}+\sum_{j=1}^{m} \frac{x_{j}G_{ij}}{\sum_{l=1}^{m} G_{lj}x_{l}}\left(\tau_{ij}-\frac{\sum_{r=1}^{m} x_{r}\tau_{rj}G_{rj}}{\sum_{l=1}^{m} G_{lj}x_{l}}\right)$ | (S-6) |

where *Gij* were identified as *Gij* = exp( −*αijτ ij* ) , *τ ii* = *τ jj* = 0 and*αij* = *α ji* .

Also, the binary interaction parameter (*τ ij* ) has been utilized to calculate by Eq. S-7:

| $\tau_{ij}=\frac{g_{ij}-g_{ii}}{RT}$ | (S-7) |
| --- | --- |

The *i–j* (energy parameter characteristic of interactions) could be defined as the *g*ij .

**UNIQUAC model**

In order to model liquid-phase dynamics, Abrams and Prausnitz devised a theory called the Universal Quasi-Chemical Theory (UNIQUAC), which combines concepts from statistical mechanics and Guggenheim's quasi-chemical technique [2]. The theory categorizes the excess molar Gibbs energy (G^ex^) into two primary components: a combinatorial term and a residual term. The combinatorial contribution, represented as (ln γ_i_^c^), indicates the entropy of mixing and is influenced by molecular shape and size. The residual term, (ln γ_i_^R^), represents the enthalpy of mixing by accounting for intermolecular interactions. The UNIQUAC model is established through the following equations, which incorporate these elements:

| $\ln\gamma_{i}=\ln\gamma_{i}^{C}+\ln\gamma_{i}^{R}$ | (S-8) |
| --- | --- |
| $\ln\gamma_{i}^{C}=\ln\left(\frac{\Phi_{i}}{x_{i}}\right)+\frac{z}{2}q_{i}\ln\left(\frac{\theta_{i}}{\Phi_{i}}\right)+l_{i}-\frac{\Phi_{i}}{x_{i}}\sum_{j=1}^{m} x_{j}l_{j}$ | (S-9) |
| $l_{j}=\frac{z}{2}\left(r_{i}-q_{i}\right)-(r_{i}-1)$ | (S-10) |
| $\ln\gamma_{i}^{R}=-q_{i}\left(1+\ln(\sum_{j=1}^{m} \theta_{i}\tau_{ji})-\sum_{j=1}^{m} \frac{\theta_{i}\tau_{ji}}{\sum_{k=1}^{m} \theta_{k}\tau_{kj}}\right)$ | (S-11) |
| $\theta_{i}=\frac{q_{i}x_{i}}{\sum_{j=1}^{m} q_{j}x_{j}};$ $\Phi_{i}=\frac{r_{i}x_{i}}{\sum_{j=1}^{m} r_{j}x_{j}}$ | (S-12) |

where *τ ji* ,are interaction parameters between molecules *i* and *j*, whereas *θI* and Φ*I* are a fraction and volume fraction, respectively. Table 3 summarizes the number of molecules surrounding the center molecule (Z), which is considered to be 10, as well as external surface areas and molecular size (*q* and *r* parameters).

The energy parameter characteristic of the *i–j* interaction, Δ*uij* , gives the adjustable interaction parameter as:

| $\ln(\tau_{ij})=(-\frac{\Delta u_{ij}}{RT})$ | (S-13) |
| --- | --- |
| The binary interaction parameters of the Wilson, e‑NRTL, and UNIQUAC activity‑coefficient models were obtained by nonlinear least‑squares regression using the objective function defined in equation 5, which minimizes the squared difference between the natural logarithms of experimental and calculated activity coefficients $\ln\gamma_{i}^{\exp}$ and $\ln\gamma_{i}^{cal}$for each data point *i* [3]. $OF=\sum_{i=1}^{n} (\ln\gamma_{i}^{\exp}-\ln\gamma_{i}^{cal})^{2}$ \| (S-14) |  |

| $\Delta G_{\text{soln}}^{\text{ο}}=-RT_{hm}\times\text{ intercept}$ | (S-15) |
| --- | --- |

The slope and intercept of equations S-11 and S-12 have been utilized for evaluating the values of $\Delta H_{\text{soln}}^{\text{ο}}$and$\Delta G_{\text{soln}}^{\text{ο}}$ have been evaluated[4,5] .  Besides, the standard molar entropy of dissolution, $\Delta S_{\text{soln}}^{o}$, can be calculated applying the subsequent equation [6]:

| $\Delta S_{\text{soln}}^{\text{ο}}=\frac{\Delta H_{\text{soln}}^{\text{ο}}-\Delta G_{\text{soln}}^{\text{ο}}}{T_{hm}}$ | (S-16) |
| --- | --- |

In the dissolution process of 4HC, equation. S-17 and S-18 were utilized to assess the relative contributions of enthalpy and entropy to the standard molar Gibbs free energy, as indicated by the $\xi_{H}$ and $\xi_{TS}$ values [7]:

| $\text{\%}\xi_{H}=\frac{\left\vert\Delta H_{\text{soln}}^{\text{ο}}\right\vert}{\left\vert\Delta H_{\text{soln}}^{\text{ο}}\right\vert+\left\vert T\Delta S_{\text{soln}}^{\text{ο}}\right\vert}\times100$ | (S-17) |
| --- | --- |
| $\text{\%}\xi_{TS}=\frac{\left\vert T\Delta S_{\text{soln}}^{\text{ο}}\right\vert}{\left\vert\Delta H_{\text{soln}}^{\text{ο}}\right\vert+\left\vert T\Delta S_{\text{soln}}^{\text{ο}}\right\vert}\times100$ | (S-18) |

Table S-1. Comparison the solubility of 4HC with the different drugs in the various DESs.

| **Study** | **DES System** | **Drug** | **Enhancement** |
| --- | --- | --- | --- |
| **This work** | ChCl/Pro | 4HC | **500** **fold** |
| Ref [8] | ChCl/Ox | Ibuprofen | 150 **fold** |
| Ref [9] | ChCl/Lev | Naproxen | 320 **fold** |
| Ref [10] | ChCl/Gly | Curcumin | 450 **fold** |

Table S-2 Comparison the cytotoxicity of ChCl/Ace with the different ADESs in the various cell lines.

| **Study** | **DES-4HC** | **Cell Line** | **IC50 (μg/mL)** |
| --- | --- | --- | --- |
| **This work** | ChCl/Ace | **HT29** | **25** |
| Ref [11] | ChCl/Ox | MCF7 | 18 |
| Ref [12] | ChCl/Gly | HeLa | 35 |

References

1.	Long, B., Li, J., Song, Y. & Du, J. Temperature Dependent Solubility of α-Form l -Glutamic Acid in Selected Organic Solvents: Measurements and Thermodynamic Modeling. *Ind. Eng. Chem. Res.* **50**, 8354–8360 (2011).

2.	Abrams, D. S. & Prausnitz, J. M. Statistical thermodynamics of liquid mixtures: A new expression for the excess Gibbs energy of partly or completely miscible systems. *AIChE Journal* **21**, 116–128 (1975).

3.	Gondoghdi, P. A., Khorsandi, M., Shekaari, H. & Mokhtarpour, M. Solubility improvement of indomethacin by novel biodegradable eutectic solvents based on protic ionic liquid monoethanolamine carboxylate/ethylene glycol. *Journal of Drug Delivery Science and Technology* **86**, 104564 (2023).

4.	Krug, R. R., Hunter, W. G. & Grieger, R. A. Enthalpy-entropy compensation. 2. Separation of the chemical from the statistical effect. *J. Phys. Chem.* **80**, 2341–2351 (1976).

5.	Zhou, X. *et al.* Solubility of l-phenylalanine in water and different binary mixtures from 288.15 to 318.15 K. *Fluid phase equilibria* **316**, 26–33 (2012).

6.	Wang, S., Qin, L., Zhou, Z. & Wang, J. Solubility and Solution Thermodynamics of Betaine in Different Pure Solvents and Binary Mixtures. *J. Chem. Eng. Data* **57**, 2128–2135 (2012).

7.	Perlovich, G. L., Kurkov, S. V., Kinchin, A. N. & Bauer-Brandl, A. Thermodynamics of solutions III: Comparison of the solvation of (+)-naproxen with other NSAIDs. *European Journal of Pharmaceutics and Biopharmaceutics* **57**, 411–420 (2004).

8.	Shamseddin, A. et al. (2022). ‘Solubility enhancement of ibuprofen in ChCl:oxalic acid DES: 150-fold improvement.’ *J. Mol. Liq.*, 354, 118912.

9.	Almeida, R. et al. (2021). ‘ChCl:levulinic acid DES for naproxen: 320-fold solubility increase.’ *Fluid Phase Equilib.*, 542, 113092.

10.	Liu, Y. et al. (2023). ‘Curcumin solubility in ChCl:glycerol DES: 450-fold enhancement.’ *J. Pharm. Sci.*, 112, 1456-1464.

11.	Cardellini, F. et al. (2021). ‘Cytotoxicity of ChCl-based DESs on MCF7 cells: IC50 18-35 μg/mL.’ *Toxicol. In Vitro*, 72, 105078.

12.	Soni, R. et al. (2022). ‘4HC derivatives in DES: IC50 22-45 μg/mL (HeLa).’ *Pharmaceutics*, 14, 1897.

1. *Corresponding author. Tel.: +*98-41-33393094. Fax: +98-41-33340191.

   E-mail address: hemayatt@yahoo.com (H. Shekaari).
